# Adult Mesenchymal Stem Cells: When, Where, and How

**DOI:** 10.1155/2015/628767

**Published:** 2015-07-26

**Authors:** Arnold I. Caplan

**Affiliations:** Skeletal Research Center, Department of Biology, Case Western Reserve University, Cleveland, OH 44106, USA

## Abstract

Adult mesenchymal stem cells (MSCs) have profound medicinal effects at body sites of tissue injury, disease, or inflammation as either endogenously or exogenously supplied. The medicinal effects are either immunomodulatory or trophic or both. When to deliver these mediators of regeneration, where, and by what delivery apparatus or mechanism will directly determine their medical efficacy. The MSCs help manage the innate regenerative capacity of almost every body tissue and the MSCs have only recently been fully appreciated. Perhaps the most skilled physician-manager of the body's innate regenerative capacity is in orthopedics where the vigorous regeneration and repair capacity of bone through local MSCs-titers is expertly managed by the orthopaedic physician. The challenge is to extend MSCs expertise to address other tissue dysfunctions and diseases. The medicine of tomorrow will encompass optimizing the tissues' intrinsic regenerative potential through management of local MSCs.

## 1. Introduction

Since the late 1980s when the technology for isolating and culture expanding MSCs was perfected and then reduced to practice in the early 1990s [1, 2], their use for clinically relevant therapies has evolved. Indeed, two very different logics have been proposed and explored. The original logic was that marrow-derived, culture-expanded MSCs, because of their multipotency, could be used in tissue engineering formats to replace injured, damaged, or diseased mesenchymal tissues [3, 4]. Although this logic was pursued for almost three decades and continues to be explored, no product or treatment is currently available. In defense of this pursuit, newer logics and scaffolds now being experimentally tested hold realistic promise for eventual success and clinical use to replace cadaveric products now used routinely.

The documentation that MSCs (perhaps all MSCs) are derived from perivascular cells, pericytes [5, 6], now explains how MSCs can be isolated from almost every tissue in the body [7, 8]. Moreover, the fact that MSCs possess the capacity to secrete immunomodulatory and trophic mediators strongly argues that their natural and normal* in vivo* function is as Medicinal Signaling Cells (MSCs) for sites of injury or inflammation [9, 10] in all of the tissues in which they are housed. Today on the website *clinicaltrials.gov* a search using “mesenchymal stem cells” in the website's search engine shows that over 500+ clinical trials are listed covering a surprisingly enormous array of clinical conditions. All of these clinical conditions have one or both of the immunomodulatory or regenerative (trophic) aspects as central components to the therapeutic intent of using MSCs.

The focus of this treatise is to take the state of knowledge, at* this point in time*, to address the medicinal use of MSCs and to attempt to identify the key parameters to consider for their optimal use in cell-based therapies. In this context, some misconceptions will be addressed since the state of detailed knowledge is relatively small compared to the exuberant expectations of the physicians and scientists consumed by the therapeutic potential of MSCs, the present author included. Thus, this paper is a report on the state of the art of MSCs and it is expected that these new, powerful potential therapeutics will evolve as we have previously witnessed when considering the changes in use and science of hematopoietic and neural stem cells in the last 50 years of their clinical and experimental exploration [11, 12].

## 2. MSCs

The realization that MSCs are derived from pericytes changes the context of considering how they arise and function* in vivo* during the body's response to both localized injury and the demand for regeneration/repair. In its simplest inception, the pericyte is released from its association with the basal lamina of the blood vessel situated in the field of injury or inflammation. This released pericyte is exquisitely capable of sensing its surrounding* milieu* and responding by becoming an MSC; this new MSC phenotype becomes activated and keyed to the detailed chemistry and dynamic changes to its local microenvironment. The activated MSCs put out a concentrated localized curtain of bioactive molecules that serves to inhibit the interrogating cells of the body's overaggressive immune system [13, 14]. This is, thus, a first-line defense against the establishment of autoimmune reactions against the injured tissue in the immediate vicinity. In coordination with this protective curtain, the MSC secretes molecules to assist in the establishment of a regenerative (not repair) microenvironment. Included in these trophic mediators are molecules that (A) inhibit ischemia-caused apoptosis; (B) inhibit scar formations; (C) stimulate angiogenesis and vessel stability; and (D) stimulate mitosis of tissue-intrinsic progenitors [15, 16].

The overall effect of locally situated, activated MSCs is to help manage the innate capacity of every tissue to regenerate itself by inhibiting the quick-fix apparatus of scar formation. It is now apparent that the immune system contributes components that not only protect injury sites from “foreign intruders” but also enhance the quick-fix aspects of fill-in with connective tissue that leads to scar. Clearly, in embryos where the immune system has not developed, scarless healing is quite normal [17]. Likewise, in neonates, the scarless regenerative capacity is substantial. As animals get larger and as they age, the vascular density in various tissues decreases and tissue regeneration, or even repair, becomes logistically different [18]. The key to the MSCs' clinical efficacy is the fact that every living tissue turns over. This means that as cellular and extracellular matrix (ECM) components expire, they are replaced by similar components. The innate regenerative capacity of a tissue is tied to this turnover dynamic. For example, the fact that bone is resorbed and fabricated in a coupled cellular mechanism allows fractures to heal at a rate directly linked to the natural, age-related ratio of fabrication to resorption, that is, rapid healing in young growing subjects and very slow healing in older, osteoporotic subjects [19, 20]. This begs the question of whether it is a youthful microenvironment (i.e., molecular) that controls turnover/repair or whether it is the cells themselves that provide the dynamic queuing.

If MSCs are, indeed, the managers of site-specific tissue regeneration, their presence, their numbers, their proper activation, and their coordinated and dynamic function can have a profound impact on injury and disease progression. The medicinal activity of MSCs is, thus, dependent on aspects of the management of the tissue and the site of injury or disease with respect to the therapeutic capacity of either endogenous or exogenously supplied MSCs. This infers that MSCs are intrinsically curative and that their therapeutic effectiveness solely depends on the “when, where, and how” of their delivery or presentation at sites of injury, disease, or regeneration.

## 3. When

At the site of any tissue injury, large or small, there is an immediate trigger to the acute inflammatory response which serves to bathe the site with molecules and cells to protect against invasion by toxic molecules or foreign organisms. This acute inflammation serves to also condition the site for either regeneration, repair, or scarring. The presence of MSCs following this initial flushing of the injury site would inhibit the intrusion of immune interrogating cells and further protect the site from the disbursement of agents that could be toxic to resident tissue cells. The activated MSCs function to inhibit connective tissue cells from pumping out massive amounts of collagen and other components that function as both the soil and the bed for scar. Thus, early in the injury response, sufficient numbers of MSCs could naturally serve to protect the injury field from degenerate events and allow regenerative repairs to be initiated. In this regard, in an aging individual with decreased numbers of MSCs, scarring would be more prominent.

Given the above logic, the “when” to deliver MSCs is after the major acute inflammation has died down, relatively early after the injury event. This could be at 48 hours after an acute myocardial infarct or by day 7 following a stroke as observed in preclinical animal models [14–19]. If the injury or disease state is chronic, multiple presentations of MSCs, say twice per week for 4 weeks (the Osiris Therapeutics, Inc., protocol for Crohn's Disease), anticipate multiple events and an extended duration of MSC exposure. In extreme cases such a heavily scarred tissue such as observed in patients with COPD or chronic asthma [21], again multiple exposures suitably spaced from one another should be required.

The issue of scarred tissue is quite complex and the age and health status of the subject are critical. Scar is a living tissue composed of massive ECM and its maintenance cells. The assumption is that scar, say in the lung, turns over. If MSCs do indeed function, either inhibiting the formation of scar or inhibiting the entrance or development of scar forming cells, then MSCs must reside at sites of scar for a considerable length of time or appear at critical intervals to inhibit scar formation or expansion while providing a microenvironment for the afflicted tissue to regenerate itself. In an animal model of asthma, multiple exposures to MSCs are required to enhance scar turnover and its eventual elimination [21].

## 4. Circulating or Mobilizing MSCs

The best data available indicates that MSCs do not circulate [22, 23]. Indeed, when MSCs were infused into the venous system of one arm only, a few MSCs could be detected right after infusion in the blood of the other arm, but none thereafter [22]. It is important to understand that if 100 million MSCs are slowly infused into the blood stream of an adult (even if all of these MSCs circulated which is improbable), the number of circulating blood cells is in such vast excess that it would almost be impossible to detect even one MSC by cell-sorting or by colony formation (MSC adhesion to culture dishes in optimal plating medium) [24].

This also begs the question as to when and if MSCs can be mobilized to sites of injury. The entire concept of “mobilization” stems from a misconception and faulty word-usage in hematology. It is commonly accepted to call the action of the drugs G- or GM-CSF as “mobilizing” because huge amounts of hematopoietic progenitors can be detected in peripheral blood samples [25, 26]. These drugs cause massive cell proliferation in bone marrow and the progeny becomes so densely packed that they push out through the sinusoids into the blood stream. This is a cell crowding event not cell-specific mobilization. Likewise, if rodents are grown in chambers of low oxygen, MSCs can be found in circulating blood consistent with an increase in blood levels of HIF-1*α* [27]. I believe the circulating MSCs are present because of numerous blood vessel breaks and the release of pericytes from their basal lamina anchorage not because of HIF-1*α* causes the mobilization of the cells.

The recent report that no circulating MSCs could be detected in various patients with chronic diseases but could be detected in patients with multiple fresh fractures does not disprove the concept that MSCs can circulate and can be mobilized [24]. The blood sample of the chronic disease patients contained no MSCs because the initiation of the chronic condition had long since passed and the micro “injury” to sustain a chronic condition is not known and difficult to time. Moreover, the sensitivity of a cell-sort or colony plating scheme is too low to detect MSCs if, indeed, they were mobilized and circulating. Again, for emphasis, the pericyte is released from its tether in the basal lamina at injury or inflammation to become an MSC that is both mobile and it can be swept into the blood stream. More basic information is required to understand these events* in situ* before we discard the notion that MSCs can be mobilized or that they circulate. The data involving SDF-1 (discussed below) could be used to argue that MSCs are motile and dock in specific regions of the vascular tree.

## 5. Where

MSCs function at sites of blood vessel damage or inflammation. That is where they need to be delivered. This can be accomplished by systemic delivery, but it is clear that these exogenous MSCs are fragile and can be eliminated almost immediately upon entering the blood stream [28]. Likewise, they can irreversibly lodge in the lung and liver [29] and, thus, never reach the tissue target. Therefore, where exogenous MSCs are introduced in the body can have a profound influence on their capacity to reach sites of recent or current injury or inflammation. This issue of “where” to infuse MSCs has been exquisitely documented by Lin et al. who introduced MSCs into mice via a carotid cutdown using a stiff catheter into the aortic arch and, thus, into the left ventricle and descending aorta whose blood flow bypasses the lung and liver for at least one full body passage [30]. These experiments were done in a mouse in which one leg was irradiated causing a marrow injury 4 hours prior to luciferase-labeled MSC infusion. The standard tail vein infusion uses one million MSCs while left ventricle infusion could deliver 10 times less yet document that the labeled MSCs did indeed dock in the injured leg marrow.

Direct injections of MSCs into synovial joints, spinal disc, or intramuscular are also being used clinically with apparent success. The most detailed study has involved a cork-screw catheter into an infarcted heart [31]. Penn and colleagues have shown that the infarcted rodent hearts released SDF-1 and that if exogenous MSCs are delivered [32, 33] within 48 hours after injury the MSCs will dock in this tissue and protect the heart from the subsequent damaging events. Importantly, if MSCs are introduced systemically on day 7, the SDF-1 is no longer being secreted and MSCs will not dock. Moreover, by using a plasmid for SDF-1 and delivering it to damaged heart, the SDF-1 subsequently produced will serve as a powerful chemoattractant for MSCs, presumably from marrow and other depots, to attract them to the injured tissue and to assist in both the protection and the recovery of the heart tissue [34, 35]. The sustained secretion of SDF-1 also holds promise for treating patients with chronic heart issues and is part of a current clinical trial (http://www.juventasinc.com/index.html).

Last, although systemic and direct injections of MSCs into afflicted tissue are in use, the introduction of MSCs into the peritoneal cavity has never been properly evaluated, especially for Crohn's disease, inflammatory bowel disease, or ailments of the abdominal region. Since the lymph tree in this cavity is so prominent, it is tempting to propose that MSCs might be highly effective if introduced into this tree. Likewise, would this tree be a useful port for systemic introduction of exogenous MSCs?

It must be emphasized that there is no quantitative information that elaborates the number of “docked” MSCs as related to a specific therapeutic outcome. The initial intravenous doses of MSCs are extraordinarily large in both rodent-disease models and in clinical trials where 1–5 million MSCs/kg are the standard doses. Moreover, although docking strategies have been employed, the efficiency of docking and the potency of MSCs are difficult to quantitate and almost impossible to relate to the composite therapeutic outcomes. As inferred above, if MSCs must dock in the damaged heart tissue and in the servicing lymph tree, the question of efficiency of docking and potency of MSCs becomes even more difficult.

## 6. How

Although clinical trials are now in play in which MSCs are mostly delivered intravenously, intramuscularly, and into the synovial joints, there are other routes of administration that are being explored. The cork-screw catheter was used to increase the needle path (creating an increase in focal injury) and to maximize the retention of MSCs in the heart and thus delivers MSCs into afflicted cardiac tissue where the MSCs not only dock in and on this newly injured tissue, but also spill out into the circulation [36, 37]. It may be that this spillage allows the MSCs to dock in the lymph system that services the heart where they may affect the local immune system (my speculation).

A very unusual, but potentially important delivery route has been published indicating that the upper sinus might be a perfect routing to the brain. Currently, intrathecal administration of MSCs is being used for patients with MS or ALS. Cells or drugs like insulin delivered to the upper sinuses are captured by a liquid stream that flows from around sensory axons of olfactory nerves up into the extracellular fluid that courses through in the brain from front to back [38, 39]. This may also be a more logical pathway for patients suffering from Parkinson's disease or MS to receive therapeutic cells including MSCs as has been published in rodents [40].

The therapeutic effect achieved by MSCs is by producing a spectrum of bioactive molecules that affect the injury site by both trophic and immunomodulatory mechanisms. The question arises as to whether, by priorly exposing MSCs to specific agents in culture, the paracrine activities could be optimized for a specific therapeutic outcome. For example, pretreatment of MSCs with IFN-*γ* protects [41] against graft-versus-host-disease (GVHD). Importantly, MSCs (unpretreated) mount an immunomodulatory assault on GVHD and two MSC products have been approved for use in children with steroid-refractory GVHD with substantially positive outcomes. Will IFN-*γ* pretreated MSCs eliminate all GVHD? This is doubtful given the complexities involved. However, if such pretreatment eliminated a sizeable proportion of GVHD upon bone marrow transplantation, this could save many lives and decrease the huge hospital costs.

Last, since cultured, exogenous MSCs are delicate and susceptible to damage upon entering the blood stream [28] or by direct injection into tissues; the encapsulation of MSCs may be a preferred route of administration with their subsequent slow release. For example, a small private company in Italy called Lipogems (for whom I currently consult) has an apparatus for treating lipoaspirate and generating 500 micron aggregates of adipocytes with MSCs trapped inside [42]. These aggregates when introduced into culture do not plate out, but MSCs can be observed to crawl out onto the plate after 4–7 days. Such autologous MSCs would appear at sites of injury after the acute inflammatory phase of their introduction and could be then highly effective. Clinical use for fecal incontinence, osteoarthritis, muscle injury, and so forth has been reported to be highly effective. Proper double-blind, placebo control clinical trials should be quite interesting for this MSC slow release and protective technology.

## 7. Who Makes the Therapeutic Molecules?

Because MSCs function medicinally at sites of injury, it is assumed that they produce a spectrum of therapeutically active molecules. But, do they? An ingenious experiment has been performed by Adonis Hijaz, MD, and his colleagues [43] (Hijaz et al., personal communication). A urinary incontinence model is generated in rodents by placing a balloon in the animals' vagina. The urethra is injured causing leakage of urine that can be quantitatively accessed by leak-point pressure. If human MSCs labeled with a fluorescent dye are introduced into the urethra, the animal is back to normal by day 4. If, on day 1, the animal is sacrificed and the urethra sectioned, laser capture microscopy can isolate tissue containing the fluorescently tagged MSCs and tissue situated next to the labeled hMSCs. In a separate injured animal, the injured tissue that has never been exposed to hMSCs can be isolated by laser capture techniques. RNA chips using purified RNA from these laser captured specimens indicate that the hMSCs are making many different molecules compared to what they originally made on the Petri dish from which they were expanded and isolated. More interesting is the fact that injured tissue situated next to the hMSC is making over 90 different molecules compared to the injured tissue that had never been exposed to the hMSC. By using both rodent specific RNA chips and human specific RNA chips, the question can be asked at that one time point: who is making the therapeutically relevant molecule the rodent host tissue or the hMSC? A more detailed temporal analysis will be needed to not only answer this question, but establish the dynamic interaction between the hMSC and the injured tissue. Having stated this question related to the source of secretion of the therapeutic molecules, the introduction of MSCs acts to inhibit scarring and stimulate* de novo* regeneration [15, 16].

The reason for reviewing the above is to emphasize the emerging theme that MSCs appear to be assisting the host tissue to maximize its* intrinsic* regenerative capacity. The local management of the immune cells and the tissue specific progenitors appears to be accomplished by very few, locally situated, and short-lived MSCs. This innate regenerative capacity of almost every host tissue has never been properly managed except, perhaps, in orthopedics where the vigorous regenerative and repair capacity of bone (maybe through local MSCs) is managed by orthopedic physician interface.

The Medicine of Tomorrow may be the management of MSCs to optimize the body's very powerful and ever-changing intrinsic regenerative potential.
